# Professional diagnostic delay in osteosarcomas of the jaws

**DOI:** 10.4317/medoral.24334

**Published:** 2020-10-30

**Authors:** Isaäc van der Waal

**Affiliations:** 1 Department of Oral and Maxillofacial Surgery/Pathology Amsterdam University Medical Center/ Academic Center for Dentistry (ACTA), Amsterdam

## Abstract

A series of 20 consecutive patients with an osteosarcoma of the jaws has been evaluated with regard to possible professional diagnostic delay. When set at an arbitrarily chosen period beyond three months, professional delay occurred in 15 patients, the mean being 21 months and the median 11 months. In five of the 15 patients a wrong diagnosis has been rendered on the biopsy specimen, being fibrous dysplasia (2x), osteoma (2x) and, in case of palatomaxillary swelling, pleomorphic adenoma (1x). In the other ten patients the initial clinicoradiographic features were misleading and apparently not indicative of a malignancy, except for one patient in whom a distinct widening of the periodontal ligament, as expressed on a periapical film, has been overlooked or not properly interpreted. It has not been possible to assess the possible influence of the delayed diagnosis on the prognosis.

** Key words:**Osteosarcoma of the jaws, diagnostic delay.

## Introduction

Approximately 5% of all osteosarcomas arise in the jaws. The incidence of osteosarcomas of the jaws (OSJ) is ≤ 1 per million population per year. There is no distinct gender preference. OSJs may occur at all ages, while OSs elsewhere in the skeleton are characterized by a bimodal age distribution with the first peak between the age of 10-14 years and the second peak after 65 years (1). The average age of OSJs is approximately 10 years higher than in OSs outside the jaws, being around 35 years (2).

There are no known etiologic factors, apart from the rare case of previous irradiation of the jaws or a genetic predisposition, e.g. in case of Li-Fraumeni syndrome.

In contrast to osteosarcomas in the long bones, pain is not a prominent feature of OSJ. In mandibular OSJ paraesthesia or anaesthesia may be an early symptom, depending on the precise location. In dentate patients loosening of teeth may be a late sign. The most common presentation of OSJ is a slowly growing, bony hard swelling. The mandible and maxilla are equally affected. Simultaneous involvement of the mandible and maxilla is exceedingly rare (3).

The radiographic features range from radiolucent to radiopaque or mixed changes. The cortical bone may be expanded or perforated. When the periosteum is involved an almost diagnostic 'sunray pattern' may be observed on an occlusal view or CT or MRI scan. More or less symmetrical widening of the periodontal ligament as observed on a periapical film, is another alarming sign (4).

Histopathologically, osteosarcoma is characterized by formation of osteoid by malignant mesenchymal cells. Also the presence of chondroid or abundant fibrous tissue may be encountered. In rare cases there is abundant formation of multinucleated giant cells.

Treatment of OSJ consists of surgical removal. The prognosis of OJSs is much better than for OSs elsewhere in the skeleton (5,6), the reported 10-year survival rates varying from 55% to 80% respectively. Different from osteosarcomas elsewhere in the skeleton there is no proven benefit of the use of (neo)adjuvant chemotherapy (6,7). There is no proven benefit from postoperative irradiation either (8).

Local failure is the main cause of death. Hematogenous metastatic spread of OSJs is much less common than in other osteosarcomas where up to 90% of the patients will develop pulmonary metastases.

Because of the low incidence of OSJ and the often uncharacteristic or even misleading clinicoradiographic features, it should be no surprise that there may be a considerable diagnostic delay. In addition, the histopathologic aspects may be misleading, particularly when assessed on biopsies. The aim of the present study was to assess the possible professional diagnostic delay in a series of OSJ patients of a single institute.

## Material and Methods

In the period 1971-2016 the records of all patients who have visited the department of oral and maxillofacial surgery of a teaching hospital in the Netherlands, and who were diagnosed with OSJ, have been reviewed. All cases of osteosarcoma have been reviewed by a National Bone Pathology Registry in the Netherlands.

Professional diagnostic delay (doctors' or dentists' delay, either in the first-line or second-line of healthcare), has been defined as a somewhat arbitrarely chosen period beyond three months after the first visit to a healthcare provider and a final histopathologic diagnosis of osteosarcoma.

## Results

**Figure 1:**
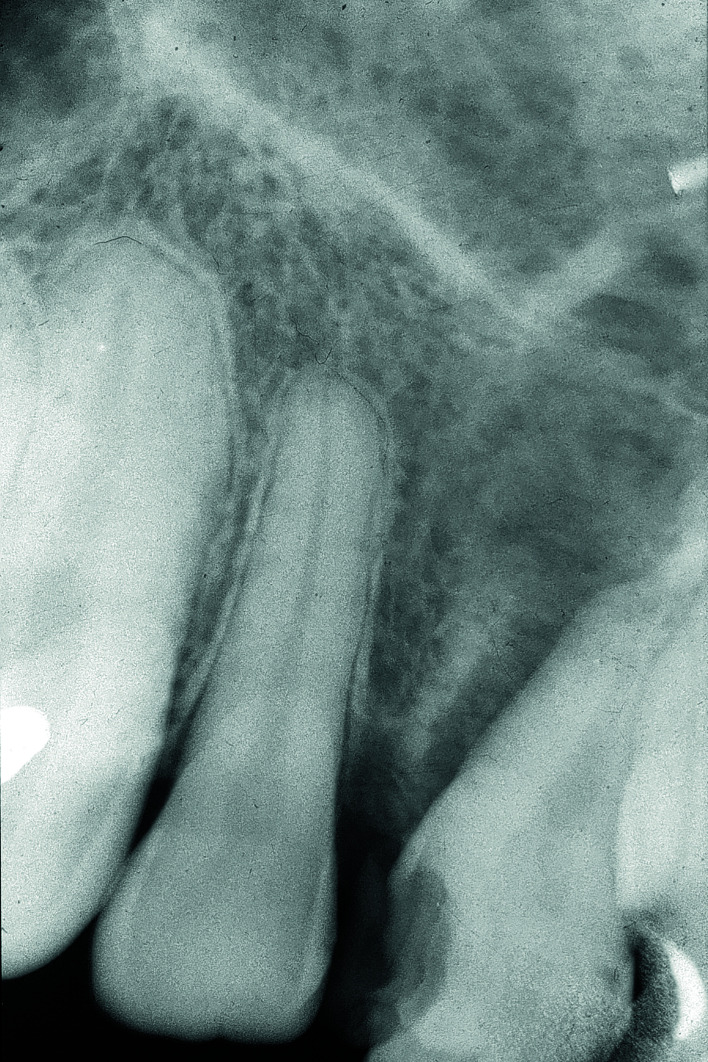
Widening of the periodontal ligament on the distal radicular surface of 11, being suspicious for malignancy, in particular for osteosarcoma.

A total number of 20 consecutive patients have been included, in whom 15 patients were initially only observed without the taking of a biopsy and/or in whom a benign histopathologic diagnosis was rendered on the biopsy specimen. The patients' data and the causes of the professional delay have been summarized in Table 1. The mean diagnostic delay amounted 21 months, the median being 11 months. In at least nine of the 15 'delayed' patients there were inconspicious or misleading clinicoradiographic features, while in one patient (#3) the suspicious widening of the periodontal ligament on a periapical film have been overlooked or not been recognized as such (Fig. 1). Probaby partly due to misleading clinicoradiographic features and, as a result, inappropriate information by the clinician, an incorrect histopathologic diagnosis of a benign lesion was recorded in five patients, being fibrous dysplasia (2x), osteoma (2x) and pleomorphic adenoma (1x). A small size or fragmentation of the biopsy specimen may have been another reason for an incorrect diagnosis in some of these five patients.

**Table 1:**
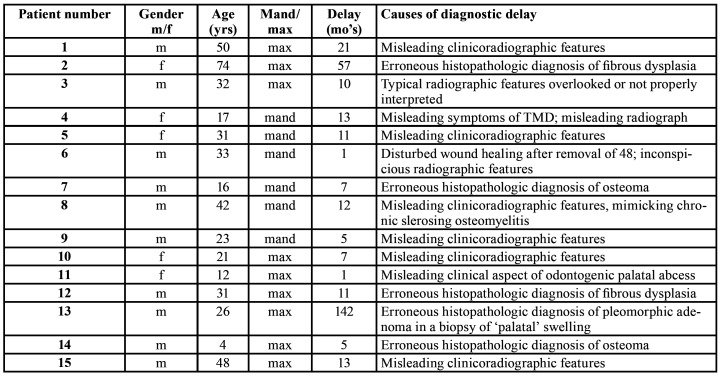
Time period and causes of professional diagnostic delay in osteosarcomas of the jaws.

In Table 2 an overview is presented of the initial management/treatment in case of professional diagnostic delay. The patient numbers in Table 2 correspond with those mentioned in Table 1.

**Table 2:**
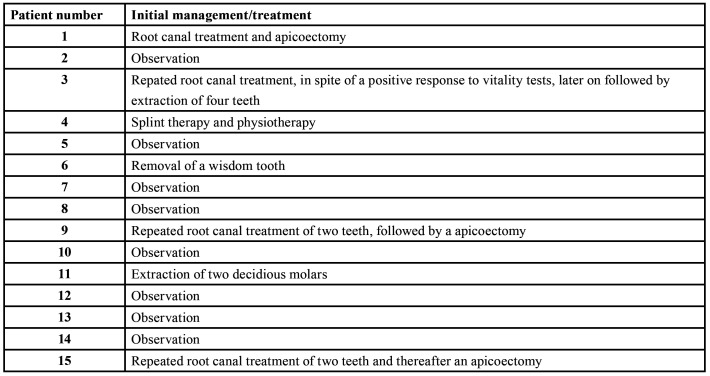
Initial management/treatment in case of professional diagnostic delay in osteosarcomas of the jaws.

Discussion and Conclusions

There are no reasons to assume that the high number of patients (15 out of 20) with a professional diagnostic delay in our study would be different from other reported series of OSJs. In the present study a cut-off point for a delayed diagnosis was set at a somewhat arbitrarily chosen period of three months. These three months included the referral time from the first-line to the second-line healthcare provider and the diagnostic work-up period in the second-line.

With regard to radiographic examination of a bony lesion of the jaws a panoramic view and, in dentate patients, one or more periapical films are the basic radiographs. In selected cases one should consider to also include an occlusal view. However, with the use of digital radiographic equipment it has become more difficult to make such a radiograph. It has been shown in a study, comprising 30 patients with different pathologies of the jaws, that the diagnosis of osteosarcoma cannot rely on radiographic characteristics alone (9). Given a suspected diagnosis of osteosarcoma additional (CB)CT-scans, SPECT-CT scans and MRIs are particularly valuable for assessment of the extent of an OSJ and its possible growth into the soft tissues.

With regard to histopathologically differentiating OSJ from fibrous dysplasia and other benign fibrous and fibro-osseous lesions, immunohistochemical expression of murine double-minute type 2 MDM2) and cyclin-dependent kinase 4 (CDK4) may be helpful (10). However, when absent, osteosarcoma can not be excluded (11). In a study based on 61 OSs no immunohistochemical marker was found to be diagnostic of OS or its subtyping (12). Unfortunately, the paraffine blocks were not available anymore to perform such immunohistochemical stains in our two patients. There are various case reports and reviews of OSJ developing from fibrous dysplasia in low grade OSs (13-15). Probably some, if not many, of the reported cases have been an osteosarcoma right from the beginning as probably has been the case in our patient #2. Re-evaluation of the initial biopsy and the clinicoradiographic documentation of this patient have not resulted in a firm diagnosis.

Particularly in case of a small or fragmented biopsy specimen it may be difficult, if not impossible, to reliably distinguish a low-grade osteosarcoma from an osteoma, as has been the case in our two patients who were initially diagnosed as osteoma. In the literature a case of osteosarcoma has been presented in which an initial diagnosis of osteoblastoma was rendered (16). In this respect also cementoblastoma could be misdiagnosed, particularly in case of a small biopsy and incomplete clinicoradiographic information. Giant-cell rich osteosarcoma is another example of possible misdiagnosis of a biopsy, being signed out as (central) giant cell granuloma.

An erroneous histopathologic diagnosis of pleomorhic adenoma in one of our patients is probaby caused by misleading clinical information ('swelling of the palate, most likely salivary gland tumor' instead of 'enlargement of the maxillary ridge'), a small size of the biopsy specimen and/or interpretation of chondroid tissue as part of a pleomorphic adenoma. In fact, there was one other such case of a histopathologic misdiagnosis in our group of 20 patients, but this has not resulted in delay.

In the present study period no patients were encountered of an erroneous histopathologic diagnosis of osteosarcoma based on the biopsy specimen.

Whether the diagnostic delay in our patients has influenced the prognosis of the patients is impossible to assess. It has not been possible to reliably record the patient delay. It is beyond the subject of the present study to discuss the medicolegal aspects that may be associated with a delayed diagnosis of OSJ. In hindsight one might blame clinicians, radiologists and pathologists from having initially rendered an incorrect diagnosis in our 15 patients.

Close interdisciplinary cooperation is a prerequisite- but not a guarentee- for a correct diagnosis of this rare type of bone tumor, as has also been emphasized for bone tumors elsewhere in the skeleton (17).
